# Luteolin attenuates *Staphylococcus aureus*-induced endometritis through inhibiting ferroptosis and inflammation via activating the Nrf2/GPX4 signaling pathway

**DOI:** 10.1128/spectrum.03279-23

**Published:** 2024-01-03

**Authors:** Shouyang Gao, Yongjian Gao, Lifu Cai, Rui Qin

**Affiliations:** 1 Department of Obstetrics, China–Japan Union Hospital of Jilin University, Changchun, Jilin, China; 2 Department of Gastrointestinal Colorectal and Anal Surgery, China–Japan Union Hospital of Jilin University, Changchun, Jilin, China; 3 Department of Gynecology, China–Japan Union Hospital of Jilin University, Changchun, Jilin, China; Jilin University, Changchun, China

**Keywords:** Luteolin, *S. aureus*, ZO-1, endometritis, Nrf2

## Abstract

Endometritis, a local inflammatory disease, has been known as the most common cause of infertility in mares. In this study, we investigated the protective effects of luteolin on endometritis induced by *Staphylococcus aureus* (*S. aureus*) and further clarified the possible molecular mechanisms. An *S. aureus*–induced endometritis model was established by the infusion of *S. aureus* into the uterus. Luteolin was intraperitoneally administered to mice 1 h before *S. aureus* treatment. The results showed that the mice of the *S. aureus* group showed severe histological changes of uterine tissues, increased myeloperoxidase (MPO) activity, and elevated TNF-α, IL-1β, and IL-6 levels. These changes induced by *S. aureus* were dose-dependently inhibited by luteolin. Furthermore, luteolin inhibited MDA and Fe^2+^ production and increased the production of GSH decreased by *S. aureus*. Luteolin prevented *S. aureus*–induced endometrial barrier disruption through up-regulating ZO-1 and occludin expression. Luteolin dramatically inhibited *S. aureus*–induced NF-κB activation. The expression of Nrf2 and HO-1 was increased by luteolin. In addition, the inhibitory effects of luteolin on *S. aureus*–induced endometritis were reversed in Nrf2 knockdown mice. In conclusion, these data indicated that luteolin protected mice against *S. aureus*–induced endometritis through inhibiting inflammation and ferroptosis via regulating the Nrf2 signaling pathway.

**IMPORTANCE:**

Endometritis is an inflammatory disease of the endometrium, which is a common gynecological disease. Up to now, there is no evidence for the protective effects of luteolin on endometritis. The purpose of this study was to investigate whether luteolin has protective effects against *S. aureus*–induced endometritis and attempts to clarify the mechanism.

## INTRODUCTION

Endometritis is an inflammatory disease of the endometrium, which is a common gynecological disease (1). If not treated properly, it may cause myositis. Patients of endometritis often have abdominal pain, irregular menstrual cycle, abnormal bleeding, and other clinical manifestations, seriously affecting the patient’s life and health (2). Inflammation has been known to play a critical role in the development of endometritis (3). At present, anti-inflammatory therapy is most commonly used for the treatment of endometritis (4). And previous studies demonstrated that inhibiting the production of inflammatory cytokines could protect mice against endometritis (5, 6).

Luteolin, a flavonoid present in natural resources, has been reported to have anti-inflammatory effects. A previous study showed that luteolin could inhibit LPS-induced acute kidney injury in mice through inhibiting inflammatory response (7). It has been reported that luteolin had protective effects against D-galactosamine/LPS-induced liver injury in mice (8). Luteolin also suppressed LPS-induced inflammatory cytokine production in cardiac myocytes (9). Luteolin has been reported to inhibit LPS-induced lung injury in mice (10). Furthermore, luteolin has been known to inhibit LPS-induced inflammatory mediator production in RAW264.7 cells (11). Up to now, there is no evidence for the protective effects of luteolin on endometritis. The purpose of this study was to investigate whether luteolin has protective effects against *S. aureus*–induced endometritis and attempts to clarify the mechanism.

## RESULTS

### Luteolin attenuates *S. aureus*–induced uterine injury

**Fig 1:**
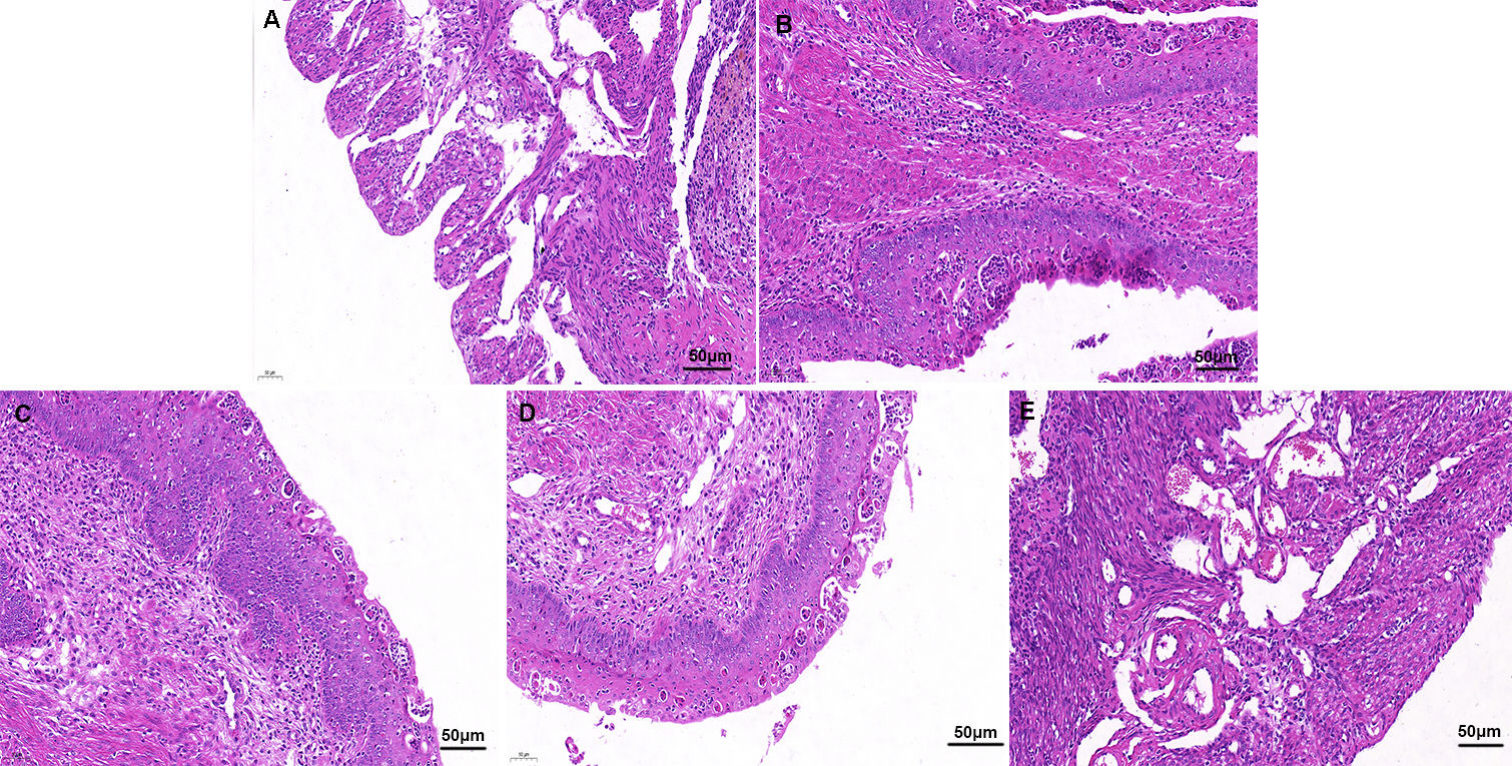
Luteolin attenuates *S. aureus*–induced uterine histopathological changes (magnification 200×): (A) control group, (B) luteolin (40 mg/kg) group, (C) *S. aureus* group, and (D–F) *S. aureus*+luteolin (10, 20, and 40 mg/kg) groups.

As shown in Fig. 1, the uterine tissues of the control group exhibited a normal structure. The uterine tissues of the *S. aureus*–treated group exhibited significant histopathological changes, including the shedding of epithelial cells and the infiltration of inflammatory cells.

### Effects of luteolin on MPO activity in *S. aureus*–induced endometritis in mice

**Fig 2:**
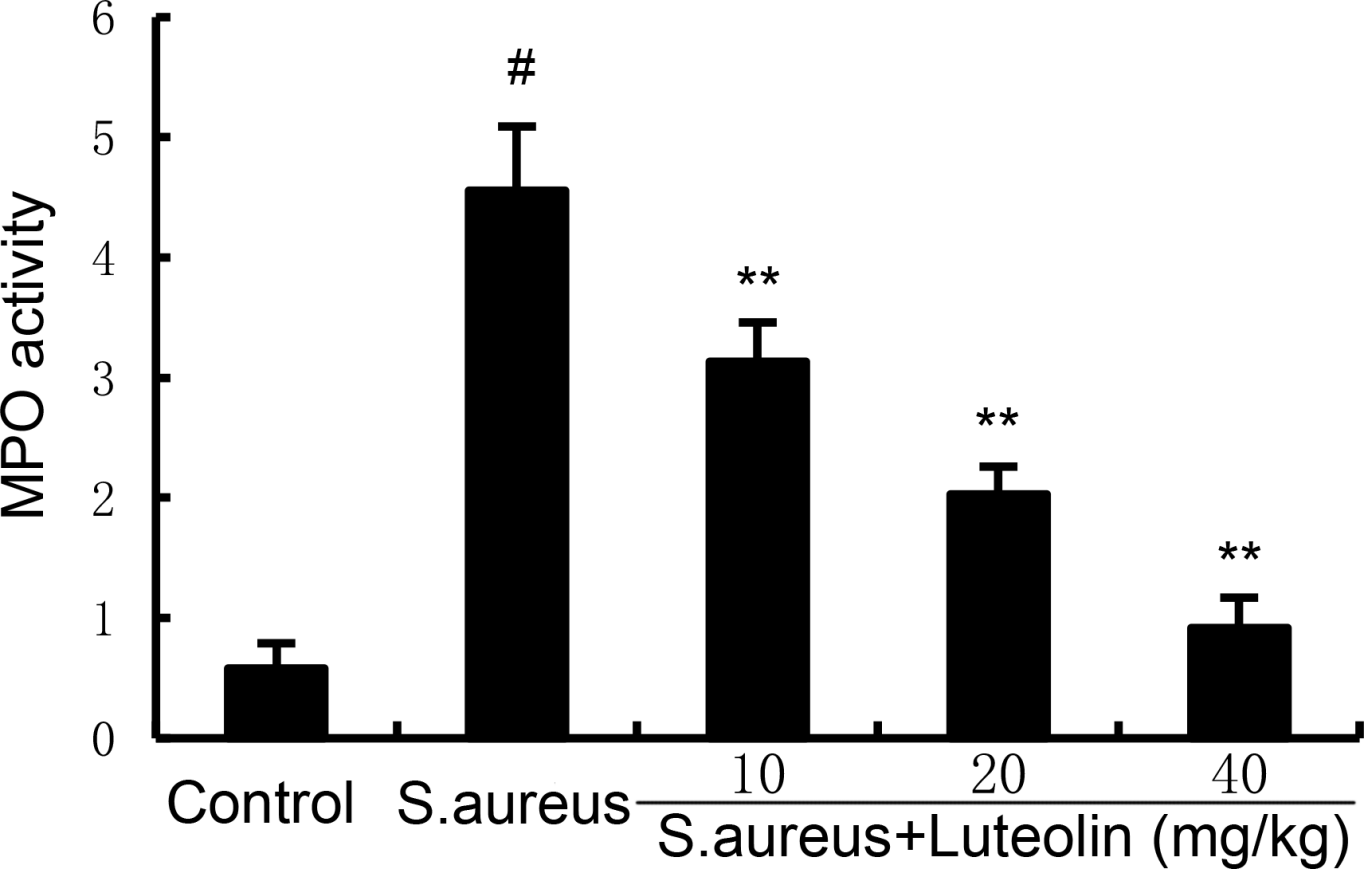
Luteolin inhibits *S. aureus*–induced MPO activity. The data of this study are presented as the mean ± SD of three parallel measurements. *^#^P* < 0.01 vs control group. ^*^P < 0.05 vs *S. aureus* group. ^**^P < 0.01 vs *S. aureus* group.

Neutrophil infiltration is a critical process in the development of endometritis. The present data showed that the MPO activity, the biomarker of neutrophils, in uterine tissues increased significantly in the *S. aureus* group. However, pretreatment of luteolin significantly reduced MPO activity in *S. aureus*–induced endometritis mice (Fig. 2).

### Effects of luteolin on inflammatory cytokine production in *S. aureus*–induced endometritis in mice

**Fig 3:**
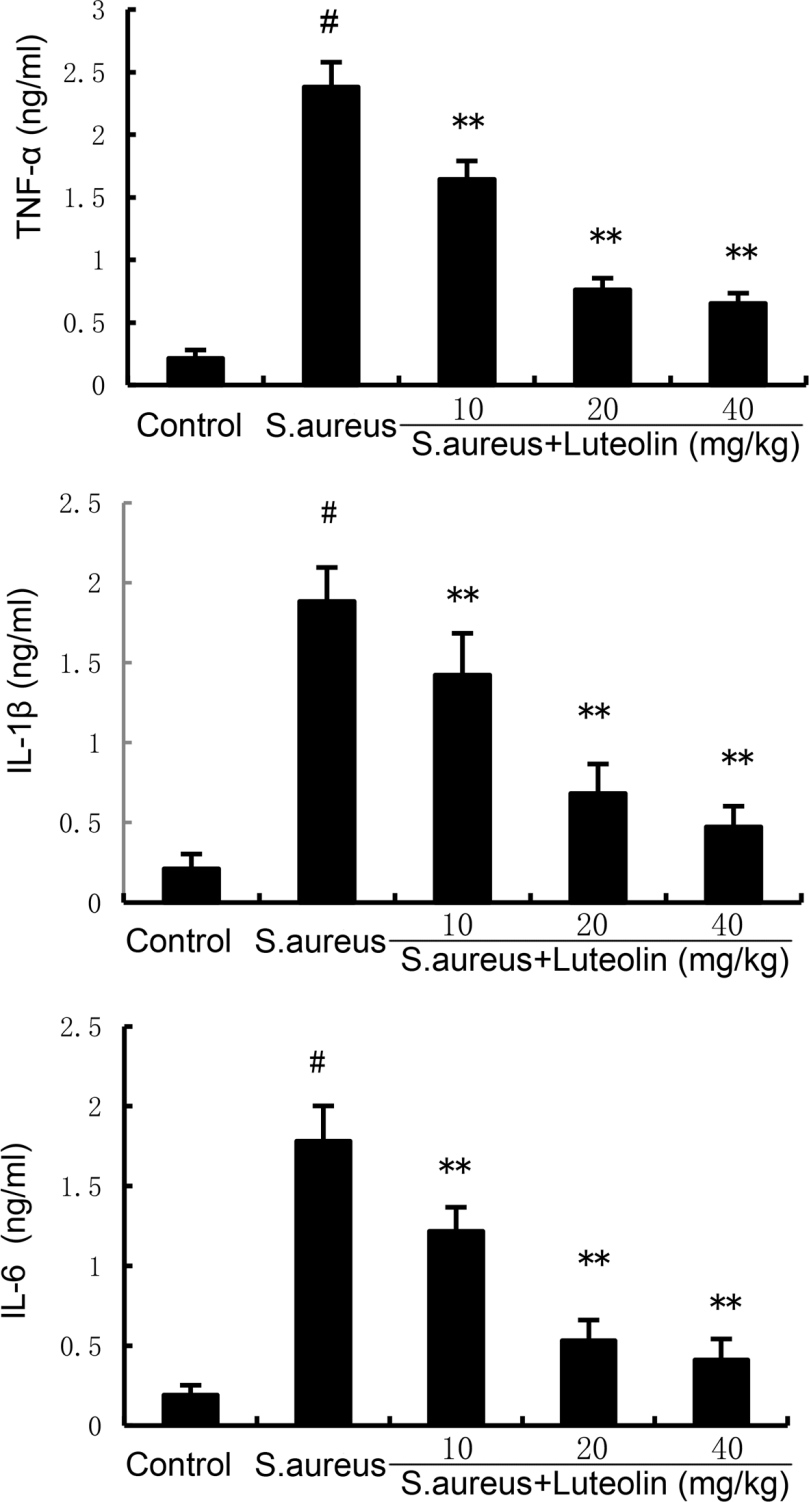
Effects of luteolin on *S. aureus*–induced TNF-α, IL-6, and IL-1β production. The data of this study are presented as the mean ± SD of three parallel measurements. *^#^P* < 0.01 vs control group. ^*^P < 0.05 vs *S. aureus* group. ^**^P < 0.01 vs *S. aureus* group.

Inflammation appears to play an important role in the development of endometritis. The present data demonstrated that the levels of TNF-α, IL-6, and IL-1β in uterine tissues increased significantly in the *S. aureus* group. However, pretreatment of luteolin significantly attenuated TNF-α, IL-6, and IL-1β production in *S. aureus*–induced endometritis mice (Fig. 3).

### Effects of luteolin on tight junction protein expression in *S. aureus*–induced endometritis

**Fig 4:**
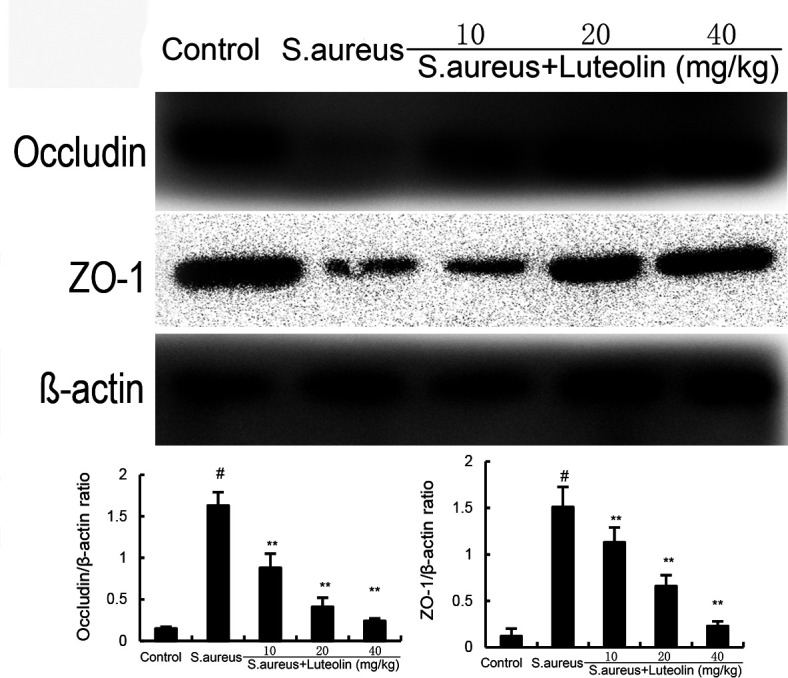
Effects of luteolin on *S. aureus*–induced tight junction expression. The data of this study are presented as the mean ± SD of three parallel measurements. *^#^P* < 0.01 vs control group. ^*^P < 0.05 vs *S. aureus* group. ^**^P < 0.01 vs *S. aureus* group.

The expression of ZO-1 and occludin in uterine tissues decreased significantly in the *S. aureus* group. However, pretreatment of luteolin significantly up-regulated the expression of these proteins in *S. aureus*–induced endometritis mice (Fig. 4).

### Luteolin attenuates *S. aureus*–induced ferroptosis in uterine tissues

**Fig 5:**
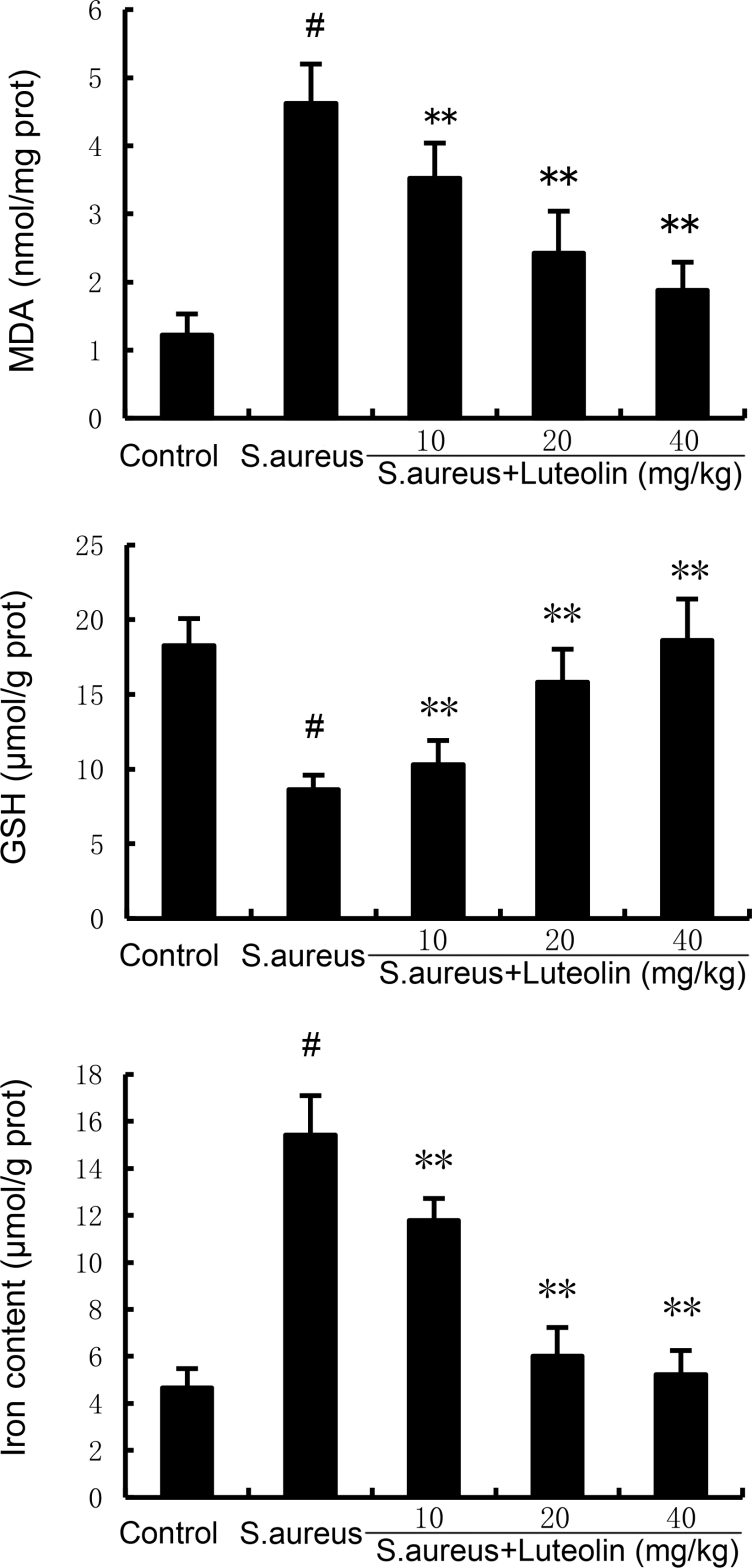
Effects of luteolin on *S. aureus*–induced MDA, GSH, and Fe^2+^ production. The data of this study are presented as the mean ± SD of three parallel measurements. *^#^P* < 0.01 vs control group. ^*^P < 0.05 vs *S. aureus* group. ^**^P < 0.01 vs *S. aureus* group.

Ferroptosis appears to play an important role in the development of endometritis. The present data demonstrated that the levels of MDA and Fe^2+^ in uterine tissues increased significantly in the *S. aureus* group. However, luteolin significantly attenuated MDA and Fe^2+^ production in *S. aureus*–induced endometritis mice (Fig. 5). Meanwhile, luteolin increased the production of glutathione (GSH (Fig. 5) and the expression of glutathione peroxidase 4 (GPX4) (Fig. 7) decreased by *S. aureus*.

### Effects of luteolin on NF-κB activation in *S. aureus*–induced endometritis in mice

**Fig 6:**
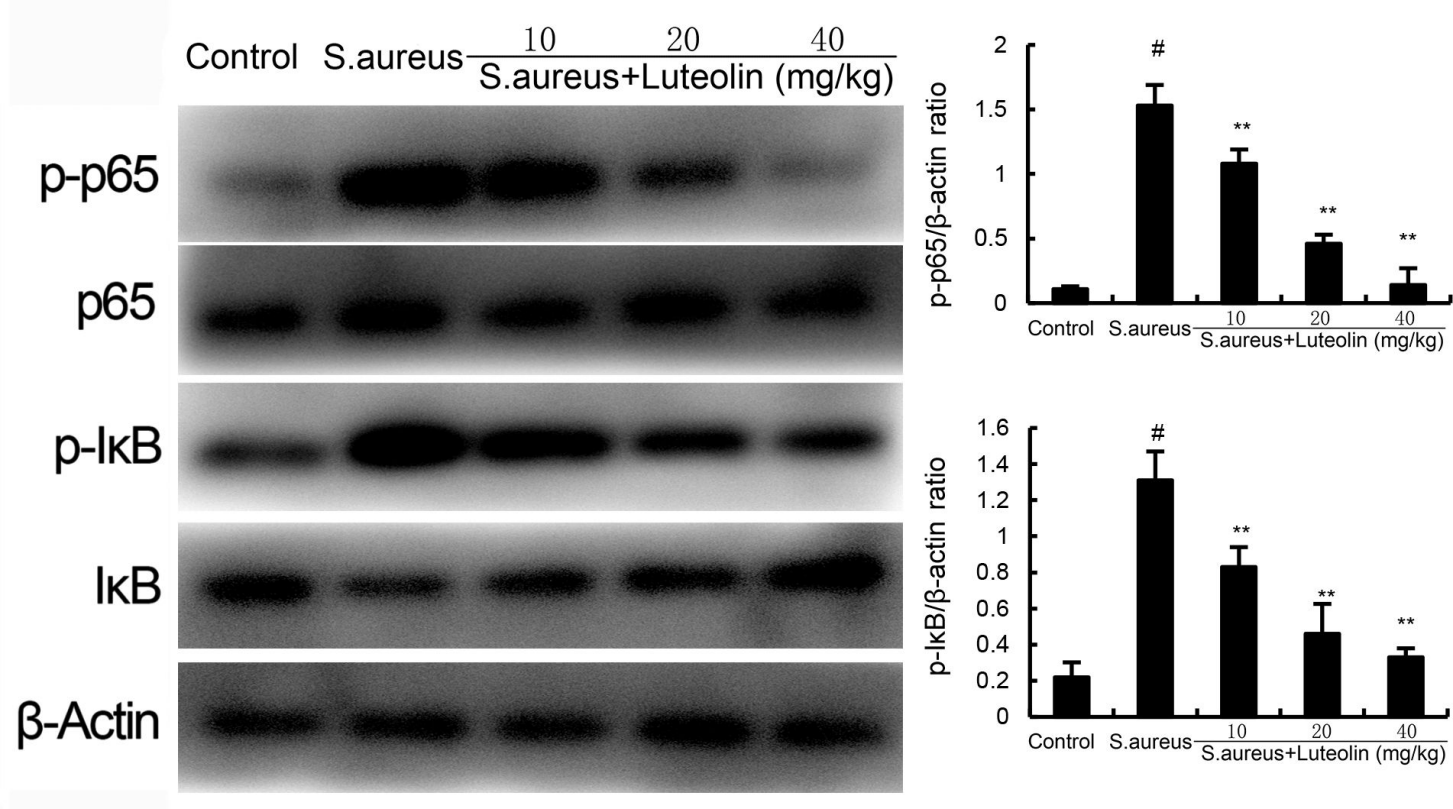
Effects of luteolin on *S. aureus*–induced NF-κB activation. The data of this study are presented as the mean ± SD of three parallel measurements. *^#^P* < 0.01 vs control group. ^*^P < 0.05 vs *S. aureus* group. ^**^P < 0.01 vs *S. aureus* group.

The protein expression of NF-κB p65 and IκBα was analyzed by western blot analysis. The present data showed that the expression of NF-κB p-p65 and p-IκBα in uterine tissues increased significantly in the *S. aureus* group. However, pretreatment of luteolin significantly inhibited the expression of NF-κB p-p65 and p-IκBα in *S. aureus*–induced endometritis mice (Fig. 6).

### Effects of luteolin on SIRT1 and Nrf2 in *S. aureus*–induced endometritis in mice

**Fig 7:**
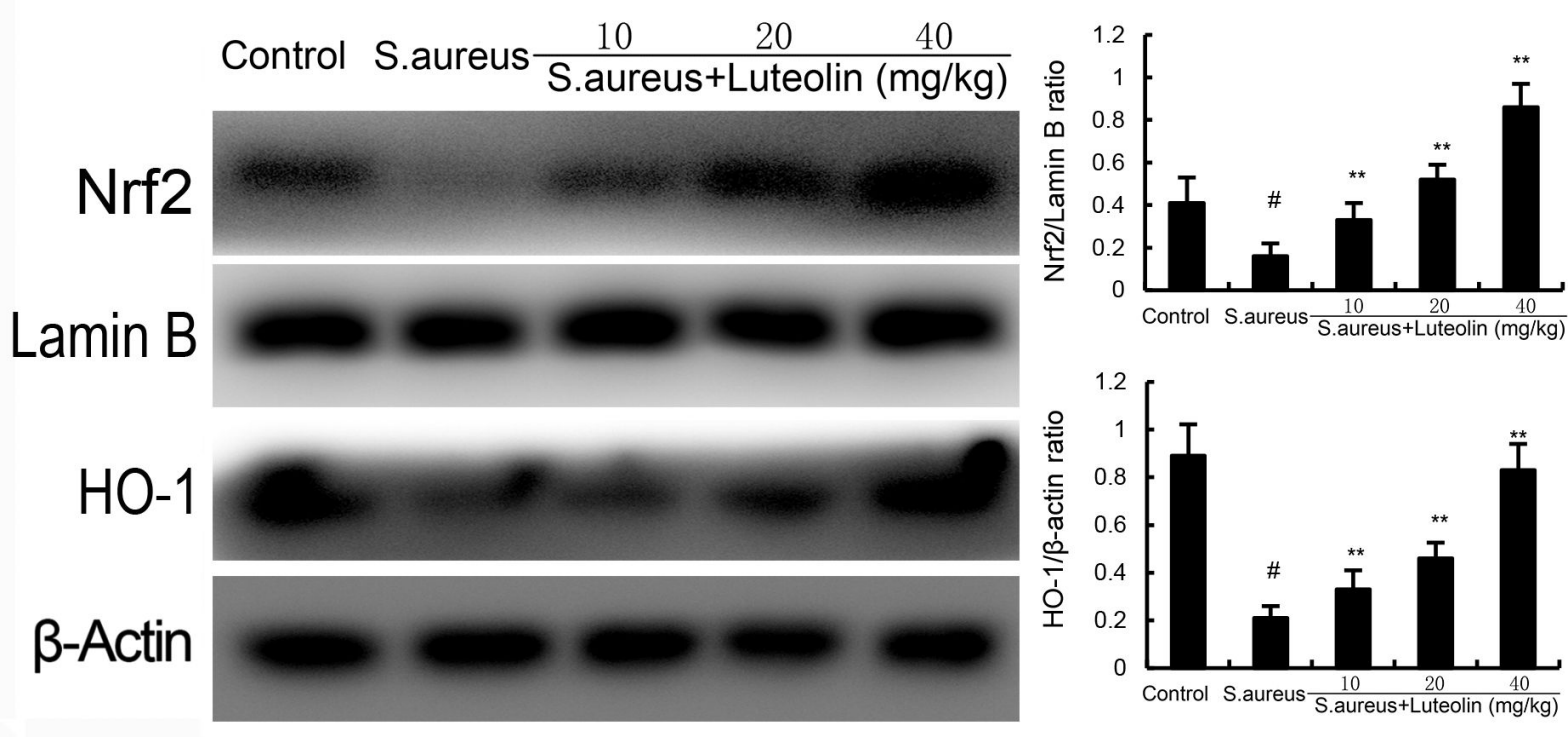
Effects of luteolin on Nrf2 and HO-1 expression. The data of this study are presented as the mean ± SD of three parallel measurements. *^#^P* < 0.01 vs control group. ^*^P < 0.05 vs *S. aureus* group. ^**^P < 0.01 vs *S. aureus* group.

**Fig 8:**
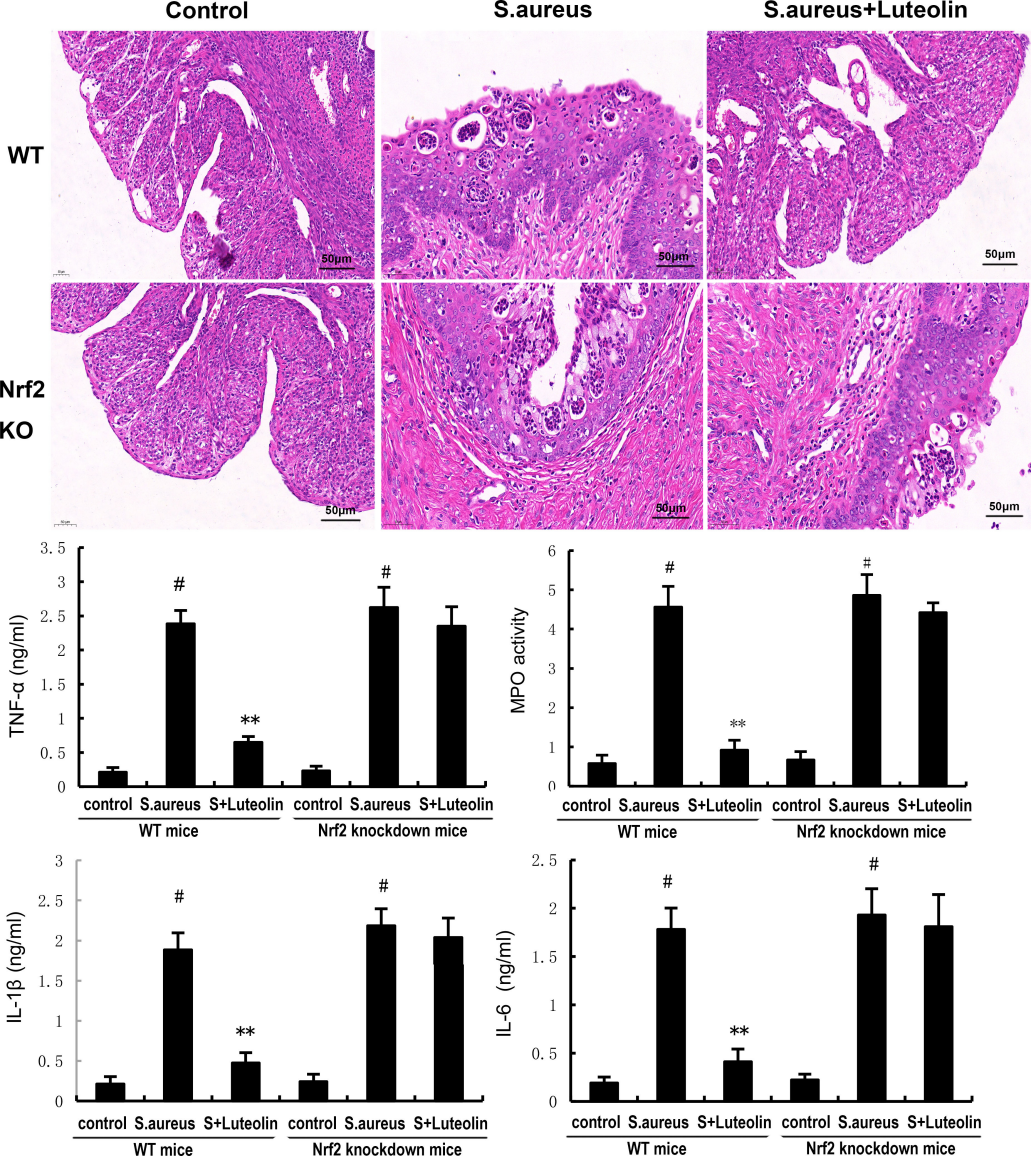
The inhibitory effects of luteolin on *S. aureus*–induced uterine histopathological changes and inflammatory cytokines were prevented in Nrf2 knockdown mice. The data of this study are presented as the mean ± SEM of three parallel measurements. *^#^P* < 0.01 vs control group. ^*^P < 0.05 vs *S. aureus* group. ^**^P < 0.01 vs *S. aureus* group.

**Fig 9:**
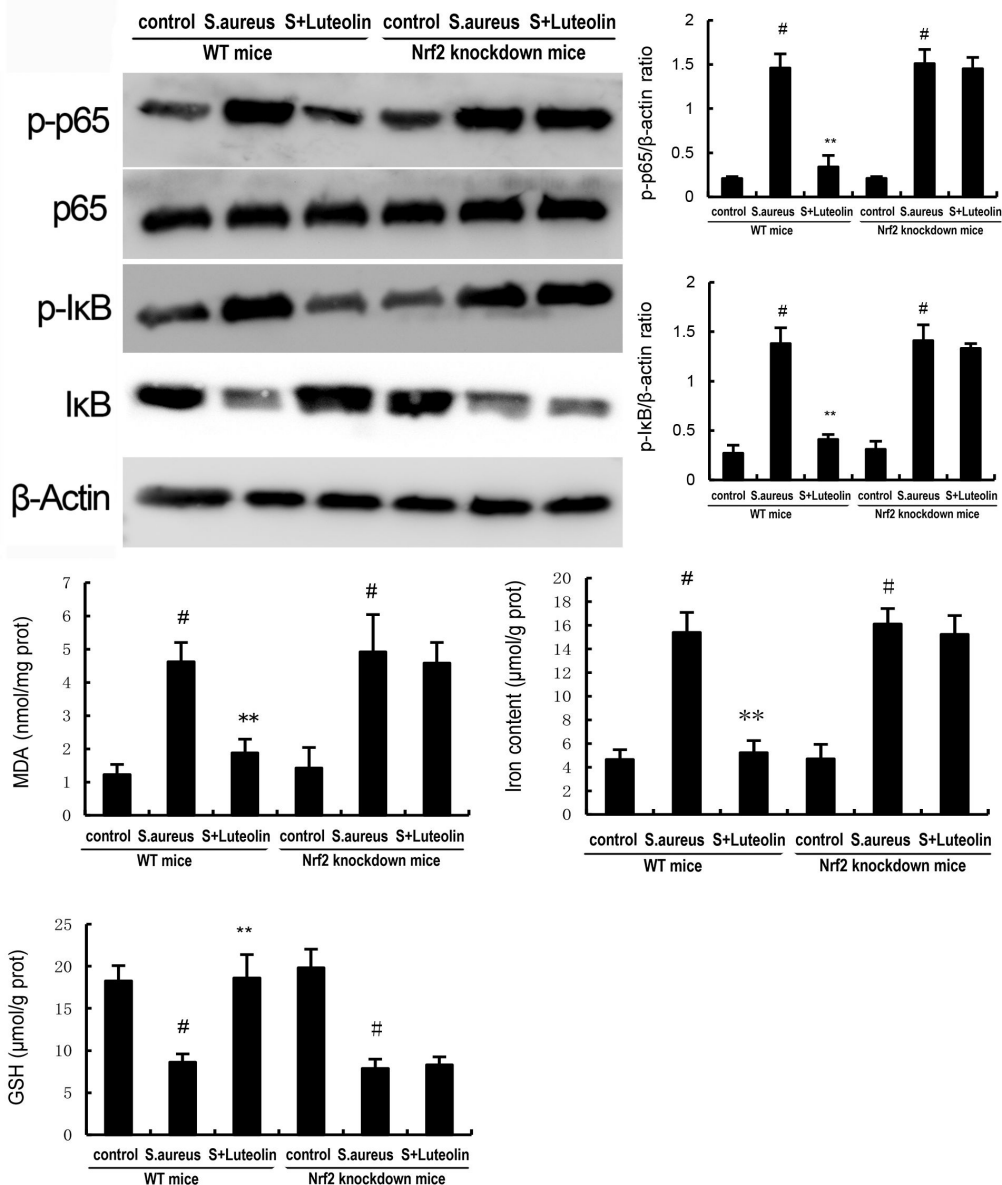
The inhibitory effects of luteolin on *S. aureus*–induced NF-κB activation were prevented in Nrf2 knockdown mice. The data of this study are presented as the mean ± SEM of three parallel measurements. *^#^P* < 0.01 vs control group. ^*^P < 0.05 vs *S. aureus* group. ^**^P < 0.01 vs *S. aureus* group.

Our data demonstrated that the expression of Nrf2 and HO-1 in uterine tissues decreased in the *S. aureus* group. However, luteolin significantly increased the expression of Nrf2 and HO-1 in *S. aureus*–induced endometritis mice (Fig. 7). Furthermore, the inhibitory effects of luteolin on *S. aureus*–induced uterine histopathological injury, inflammatory cytokines, and NF-κB were abolished in Nrf2 knockdown mice (Fig. 8 and 9).

## DISCUSSION

Inflammation has been known to be involved in the pathogenesis of endometritis (12). In the present study, we detected the protective effects of luteolin on endometritis. Luteolin was found to attenuate uterine histopathological changes and inflammatory response. The results suggested that luteolin protected against *S. aureus*–induced endometritis through inhibiting inflammatory response.

Neutrophils play an important role in the elimination of uterine infection (13). The stimulation of these neutrophils (present in the uterus and those flowing in with blood) by bacteria leads to the increased secretion of inflammatory cytokines (14). In the present study, we found that luteolin significantly suppressed *S. aureus*–induced MPO activity, which indicated luteolin inhibited *S. aureus*–induced neutrophil infiltration.

*S. aureus* is one of the most frequently isolated bacteria that cause endometritis (15). It could activate the TLR2 signaling pathway, which leads to the activation of NF-κB and inflammatory cytokine production (16). Increased inflammatory cytokines TNF-α, IL-6, and IL-1β were observed in patients with endometritis (17). In an *S. aureus*–induced endometritis model, these inflammatory cytokines were also observed in uterine tissues (18). Recently, a large body of studies demonstrated that the inhibition of these cytokines could protect mice against endometritis (19). We found luteolin dose-dependently inhibited *S. aureus*–induced TNF-α, IL-6, and IL-1β production. To clarify the mechanism of luteolin, *S. aureus*–induced NF-κB activation was measured. We found that luteolin significantly inhibited *S. aureus*–induced NF-κB activation.

Ferroptosis is one of the forms of regulated cell death, characterized by morphologically specific manifestations such as mitochondrial shrinkage and reduced number of cristae (20). The main mechanism of ferroptosis is intracellular iron overload and lipid peroxidation caused by the imbalance of redox homeostasis. GPX4 is a key antioxidant enzyme that inhibits lipid peroxidation, and its role requires GSH as a substrate (21). Therefore, ferroptosis is also characterized by a decrease in the expression of GSH and GPX4 antioxidant system factors. Previous studies demonstrated that inhibiting ferroptosis could alleviate bacteria-induced endometritis (22). In this study, we found that luteolin increased the production of GSH and the expression of GPX4 decreased by *S. aureus*, suggesting that luteolin could suppress *S. aureus*–induced ferroptosis.

Nrf2 is one of the important transcription factors that can enhance the antioxidant defense ability of the body (23). It has been confirmed by a large number of studies that its functional mechanism is through the activation of nuclear translocation, thereby promoting the expression of antioxidant enzymes such as GPX4, heme oxygenase-1, and quinone oxidoreductase-1 in the nucleus (24). Recently, it has been reported that Nrf2 could regulate inflammation and oxidative stress (25). Nrf2 has the ability to increase the resistance to oxidative stress. A previous study showed that Nrf2 could suppress oxidative stress in neuronal cells (26). Also, Nrf2 could protect against doxorubicin-induced cardiotoxicity via inhibiting oxidative stress (27). Studies demonstrated that the activation of Nrf2 could inhibit ferroptosis (28). Our data demonstrated that luteolin significantly increased the expression of Nrf2 and HO-1. And the protective effects of luteolin on *S. aureus–induced* endometritis were prevented when Nrf2 was knocked out.

In conclusion, the data of this study demonstrated that luteolin significantly inhibits *S. aureus*–induced uterine histological changes, inflammatory cytokine production, and ferroptosis. The mechanism was through regulating the Nrf2 signaling pathway.

## MATERIALS AND METHODS

### Reagents

Luteolin (purity >98%) was purchased from Sigma (St. Louis, MO, USA). The antibodies for NLRP3, ASC, caspase-1, and β-actin were obtained from Santa Cruz (TX, USA). The antibodies for Nrf2, NF-κB p65, NF-κB p-p65, IκB, and p-IκB were purchased from Cell Signaling Technology (Beverly, MA, USA). ELISA kits were purchased from BioLegend (California, USA).

### Experimental groups

Female C57BL/6J mice were provided by the animal center of Jilin University. Nrf2-knockout (Nrf2^−/−^) mice on a C57BL/6J background were purchased from the Model Animal Research Center (Nanjing, China). Sixty mice were divided into five groups: (i) control group: the mice received equal amounts of vehicle; (ii) *S. aureus* group: the mice received 50 µL of *S. aureus* (1 × 10^7^ CFU per 50 µL PBS) to each side of the mouse uterus (29); and (iii–v) *S. aureus*+luteolin (10, 20, and 40 mg/kg) groups: the mice were given luteolin (10, 20, and 40 mg/kg) intraperitoneally 1 h before *S. aureus* treatment. Twenty-four hours later, the mice were sacrificed, and the uterine tissues were collected for subsequent experiments. All the animal experiments were approved by the Ethical Committee of Jilin University.

### Histological examination

The uterine tissues were collected, washed three times using PBS, and fixed in 4% paraformaldehyde–phosphate-buffered saline. After dehydrating, the paraffin-embedded sections (5 µm) were stained with hematoxylin and eosin.

### ELISA assay

The levels of TNF-α, IL-1β, and IL-6 in uterine tissues were measured by ELISA kits (BioLegend, California, USA) according to the manufacturer’s protocol.

### MPO, MDA, GSH, and Fe^2+^ assays

The uterine tissues were homogenized, and the supernatants were collected. The MPO activity, MDA, GSH, and Fe^2+^ contents in uterine tissues were measured by the detection kits purchased from Nanjing Jiancheng Bioengineering Institute (Nanjing, China).

### Western blot analysis

The proteins (40 µg in each well) were fractionated on 12% SDS-PAGE. Then, the proteins were transported onto nitrocellulose membranes. The membranes were then blocked for 2 h with 5% nonfat dry milk and probed with Nrf2 and NF-κB (p65, p-65, IκBα, and p-IκBα) signaling pathway antibodies. Finally, the membranes were probed with the secondary antibody (1:5,000) and developed with an enhanced ECL chemiluminescence reagent (Thermo, Massachusetts, USA).

### Statistical analysis

The data were expressed as means ± SD. Statistical analysis of the data was made using one-way ANOVA or Student’s *t*-test. A *P*-value <0.05 was considered to be significant.

## Data Availability

The raw data supporting the conclusion of this article will be made available by the authors, without undue reservation, to any qualified researcher.
